# Intra aortic balloon pump: literature review of risk factors related to complications of the intraaortic balloon pump

**DOI:** 10.1186/1749-8090-6-147

**Published:** 2011-11-02

**Authors:** Haralabos Parissis, Alan Soo, Bassel Al-Alao

**Affiliations:** 1 Cardiothoracic Department, Royal Victoria Hospital, BT 12 6BA, Belfast, UK

**Keywords:** intra aortic balloon pump, IABP, complications by the use of intra aortic balloon pump

## Abstract

The increasing use of the intra aortic balloon pump is attributed to the relatively easy percutaneous insertion and the low threshold of use over the past few years, especially in elderly patients with multi-vessel diseases and an affected ejection fraction.

Unfortunately, the clinical assessment of the complications associated to the use of this supporting device, underestimates the frequency of such complications.

This report has looked at the current literature and attempt to identify incremental risk factors related to the development of adverse effects during support with an intaaortic balloon pump.

The paper concludes that in contrary to early reports, newer studies have shown that complications following intraaortic balloon pump treatment, is decreasing. Moreover the literature suggests that the thrombosis and infective complications are relevant to the duration of the pump treatment, while the ischemic problems of the limbs are mostly linked to the atherosclerotic status of the common femoral artery.

## Introduction

The complication rates when using an intra aortic balloon pump are high and may account for up to 50% according to Harvey et al [1], with an average 20-30% [2,3].

Rates depend on the definition of complications as well as on whether the data are collected in advance or not. Certain researchers argue that clinical studies may significantly underestimate the actual frequency of complications. The high occurrence of ischemia in certain studies is possibly related to the lower mortality rate. On the contrary, the low occurrence of ischemia in some other studies, may be possibly linked to the fact that the patients died following a cardiogenic shock before the development of the clinical diagnosis of vascular complications.

Thrombocytopenia is currently the commonest complication occuring in 50% of the patients followed by fever in almost 40% of the cases [2]. Bleeding is also common with aorto-iliac artery injury and dissection, thromboembolism, distal leg ischaemia and balloon entrapment-rupture, to occur less frequently. Despite this, over 70,000 IABP insertions are undertaken annually in the United States alone, with an incidence of between 5-10% amongst all patients undergoing cardiac surgery.

### Issues related to the "actual incidence" of the development of complications

Various researchers have reported only the clinically occurring morbidity during IABP treatment, while others have defined the complications in a way that encompasses all incidents, even those that have been entirely solved.

Moreover, some researchers have moved even further to include incidents where the causal role of the treatment with IABP was not clear. In a perspective study, Alderman et al [4] note limb ischemia in 42% of the cases where balloon pump was inserted.

Similarly, A. Kantrowitz et al have examined the cases of 733 patients from 1967 through to 1982 and found a total complication rate of 45% [5].

Bregman et al [6] suggest that morbidity resulting from treatment with balloon pump may go unnoticed. The main reason for that can be the short survival time after treatment with IABP might or the overall unanimity concerning the exclusion of patients on whom the insertion of intra aortic balloon pump had failed (Alpert et al) [7].

Alvarez et al [8] have retrospectively followed-up 303 patients over a decade and reported a total number of 99 patients who had complications (32.6%). However, some straightforward severe clinical indications, as in loss of pulse, were not considered to be a complication. Moreover, the paper does not make clear whether complications after failed femoral attempts (7.5%) have been included in the study.

Goldberg et al [9] argue that complication rates after failed attempts are possibly equal or higher than those of the patients with a successful IABP insertion. This is what Riaz et al [10] have also underlined. They reported a higher consequential complication rate in patients that underwent unsuccessful surgical interventions.

The fact that injuries resulting from the activity of the intra aortic balloon pump could go clinically undiagnosed despite their severity has been mentioned in an important paper by Isner et al [11]. They re-examined a total of 45 necropsy patients, who had undergone a placement of intra aortic balloon and died within 105 days after the balloon insertion.

Nine patients had a dissection of the aortoiliac axis and in none of them was the dissection suspected before their death. Insertion in 4 of the 9 patients was performed "resistance-free". In one of the 3 patients that presented arterial perforation no complication was manifested from the insertion of the balloon. Two of the 3 patients that had developed intravascular thrombosis no clinical suspicions were raised before death. Three patients presented clinically silent arterial emboli. They concluded that out of the 20 complications (in 16 patients) only 4 (20%) had been suspected before death.

### Literature review of the risk factors

#### Age

In theory, atherosclerosis develops parallel with the aging process and consequently one could conclude that the effect of vascular complications is higher in older ages. However, this speculation is an object of dispute throughout the relevant literature.

Gottlieb et al [12], undertook a multivariate analysis of risk factors in order to identify the patients at high risk for IABP related complications. They concluded that advanced age was correlated to unsuccessful insertion attempts, but it was found that it had nothing to do with the occurrence of major vascular complications, in a retrospective study in which 206 consecutive patients underwent an attempt of insertion of intraoartic balloon.

Therefore, this is not a significant risk factor.

Goldberger et al [9], mentioned their experience with 112 consecutive patients that underwent IABP counterpulsation. 11 out of 40 patients (27.5%) aged over 70 years presented complications whereas only 12 out of 72 (16.7%) patients aged less than 70 years had complications.

#### Gender

The smaller size of the femoral artery justifies the higher complication rate in women according to some researchers, such as Collier et al [13] and Beddermann et al [14]. Moreover, the same researchers concluded that men without peripheral vascular disease had lower incidence of complications related to the intra aortic balloon pump (IABP).

Shahian [15] reported that the gender was the most strongly related variable to morbidity associated with intra aortic balloon pump.

A univariate analysis of a group of 249 patients in a prospective study performed by Funk et al [16], revealed that the female gender is a significant predictor for the development of major complications. According to the same researchers, women with peripheral vascular disease and diabetes constitute the group being at the highest risk to present complications associated with the intra aortic balloon pump (83%).

#### History of peripheral vascular disease

Peripheral vascular disease is a risk factor for adverse outcome.

Pace et al [17], reexamined 104 cases of patients who had been treated over a period of 6 years. Eleven (47%) of the 23 patients with peripheral vascular disease suffered arterial complications. Iverson et al [18], examined 395 cases of patients that required IABP support. Out of 72 patients who sustained complications, 29 (40%) had a history of peripheral vascular disease.

In a retrospective study assigned to Kvilekval et al [19], 144 cases of patients that were treated with IABP were re-examined. Patients were divided into two groups. The first group had 20 patients with a history of peripheral vascular disease. The second group had 124 patients, without such a history. Twelve severe complications were observed in the first group (60% of the total 20 insertions) and seven in the second group (5% of the total 133 insertions). Moreover, the researchers examined the nature of the complications in both groups. They noticed more frequent embolic, obstructive and "technical" complications in the first group.

Alderman [4 and Skillman [20] have calculated that patients suffering from peripheral vascular disease run 1.3-1.9 higher risk to develop limb ischemia compared to patients without a history of vascular disease.

#### Insertion methods

Contradictory views have been published throughout the relevant literature.

Alcan et al [21] compared the conventional surgical and percutaneous insertion of intra aortic balloon pump in 151 cases of patients. Percutaneous insertion was attempted in 51 patients, while surgical insertion in 100 patients. The success rate in the percutaneous group was 90.2% (46 of 51) and 90% in the second group (90 of 100). The severe complications rate was found to be almost equal for both techniques (15.2 in the first group and 15.6 in the second group).

Harvey et al [1], after assessing 89 consecutive patient cases concluded that the rate and severity of complications of the percutaneous insertion of intra aortic balloon pump is similar to those of the conventional insertion of an IABP. Twenty-three patients sustained major complications and 14 sustained minor ones. Also, McCabe et al [22], after reviewing 82 patients who underwent surgical placement of an intra aortic balloon pump in the femoral artery, he mentioned a total complications rate of 23%.

On the contrary, Iverson et al [18] associated the percutaneous placement with significant decrease in complications. They reexamined 395 cases that required hemodynamic support with an intra aortic balloon. The following three methods of insertions were applied: surgical cut down, percutaneous approach and intraoperative placement in the ascending aorta. The insertion method proved to be an important factor regarding the occurrence of vascular complications. Patients in whom the intraaortic balloon pump was placed through surgical cut down had almost twice as many complications as those patients who had percutaneous placement (32 versus 19%). Despite the fact that the specific paper defends the percutaneous placement, it did not manage to explain the final outcomes and also failed to identify the complication rate for the group of patients whose balloon was placed intraoperatively in the ascending aorta.

Other studies have shown an increase in the complications associated with the balloon when employing the percutaneous approach. In a multivariate risk factor analysis Sanfelippo et al [23] concluded that the percutaneous insertion approach was associated with double number of major complications. No serious explanation has been formulated till now on this issue. However, Goldberg et al [9] explain this contradiction by suggesting that when removing a surgically placed intra aortic catheter, a Fogarty embolectomy is performed in an attempt to remove the thrombotic material.

The transthoracic intra aortic balloon pump insertion is said to be associated with a decreased rate of vascular complications of the lower extremes. This has been used as the basis for the speculation that following a treatment with an intra aortic balloon, there is high morbidity when placing a balloon percutaneously. McGeehin et al [24] reviewed 39 patients who underwent transthoracic support with intra aortic pump. Five patients sustained complications possibly associated with the procedure. Complications included mostly neurological damage and mediastinal infection.

Hazelrigg et al [25] published a retrospective review of 100 cases of transthoracic pumps. They also studied 55 cases of patients that had been placed an intra aortic balloon through the femoral vein. They mentioned a zero morbidity rate attributed to lower limb ischemia in the first group, while the rate was 16.3% in the second group. Although the duration of the balloon treatment for these two groups is not available for comparison, one could speak unfavourably for the percutaneous insertion due to the huge limb ischemic effect in the second group. The neurological incidents due to thromboembolism (resulting from in vivo formation of a clot or from managing the atherosclerotic aorta, or gaseous emboli) or hypotension, were reported to be similar in the two groups.

#### History of diabetes mellitus

Vascular complications and wound botulism are higher in patients suffering from diabetes compared to non-diabetic people.

In the framework of a comprehensive research, Wasfie et al [26] investigated 733 cases of patients undergoing intra aortic balloon pump treatment. One hundred thirty two of the patients were diabetics, 51 were on special diet, 46 were taking hypogylcemic drugs per os and 35 were insulin dependent. Vascular complications associated with the IABP method occurred in 35% of the insulin-dependent diabetics, in 18% of the "other types of" diabetics and in 14% of the non-diabetic patients. Wound infection was 37, 22 and 25% respectively. Finally, the premature mortality rate was similar for the diabetic and non-diabetic people.

However, the results of the univariate regression analysis of 46 cases by Funk et al [16] showed that diabetes mellitus was closely associated with lower limb ischemia. They concluded that a man without peripheral vascular disease or diabetes mellitus had 29% chances to sustain lower limb ischemia, while a woman with diabetes mellitus and peripheral vascular disease had 83% chances to present with this specific complication.

#### Several other risk factors

Low cardiac output, cardiogenic shock, hypertension, smoking, obesity, inotropic support, increased systemic vascular resistance, ankle-brachial pressure index < 0.8.

All these factors have been blamed for being etiologic risk factors for the occurrence of complications. In the aforementioned paper by Funk et al [16] obesity, low cardiac output, increased systemic vascular resistance and the use of inotropic support have been correlated to the incidence of vascular complications. Vascular complications were also higher in patients with hypertensions (27 v. 20%).

#### Sheathless balloon insertion: Effect on vascular complications

Apart from the percutaneous placements of the intra aortic balloon, another two major steps have been the reduction of the balloon's diameter and the sheathless technique.

Although there has been recent progress in further reduction of the catheter's internal diameter with a balloon at 7.5Fr, there has been no similar reduction in the outer diameter of the catheter, which is still 11 Fr. The use of a sheathless balloon overcomes this obstacle. As far as the impact of this technique on vascular complications, Nash et al [27] mention a limb ischemia rate of 10%. Furthermore Erdogan et al [28] recommend the use of the sheathless insertion technique in high-risk patients, particularly those with peripheral vascular disease and diabetes.

#### The duration of the IABΡ treatment

In the series of Isner et al [11], 12 of the 20 complications (60%) occurred at the time of the balloon's insertion. Easy insertion of the balloon does not exclude the possibility of severe complications. In this series, 9 patients were identified with aortoiliac dissection, however in four of the nine patients, the insertion of the device was free of resistance or other difficulties.

Six patients presented thromboembolism when the balloon was in action and one presented with focal sepsis.

Contrary to the above, Hedenmark el al [29] analyzed 90 cases of patients requiring IABP treatment over a 5-year period and concluded that most of the complications occurred at the time of the counterpulsation; specifically in 14 patients (15.55%). However, these researchers failed to describe the anti-thombotic status during the operation of the balloon and to mention whether the vascular complications were a result of thrombosis or plaque rupture, thus contributing to embolic consequences.

The fact that there is a dispute among researchers regarding the relation between the duration of the IABP treatment and the occurrence of complications does not come as a surprise. The duration of use was the only variable associated with the balloon and which could be considered to be significant (p = 0.051) in the papers published by Funk et al [16]. In patients with limb ischemia, the mean counterpulsation was 49.5 hours compared to a mean value of 42.5 hours in patients without ischemia.

Freed et al [30] analyzed 733 consecutive patients in the period from 1967 to 1982, paying special attention to the complications during the prolonged circulatory support using an intra aortic pump. Twenty seven patients were supported for over 20 days. They concluded, that complications were more frequent in patients of prolonged support.

Vascular complications were 37% compared to 15% in those who were supported for less than 20 days. Similar conclusions were reached by Iverson et al [18], and more specifically, patients requiring support through a balloon for over 60 hours presented 1.5 times higher complication rate than those that required support for less than 60 hours (32 compared to 21%). McEnany et al [31] also agree that time is a significant factor regarding the occurrence of complications associated with the balloon. They stated that 17.7% of the patients that survived, subsequently presented some complication associated with the balloon, and a complication associated with the balloon has occurred in 5.9% of the patients that died while the balloon had not yet been removed. The highest complication rate of 23.2% occurred in patients from whom the balloon had been removed and later they died. In these patients, the mean duration of treatment via an IABP was longer (average > 6 days). They concluded that septic and thrombotic complications are directly related to the duration of the support using an IABP.

Moreover, even the early vascular complications (claudication, nerve paresis and delayed vascular and neurological symptoms) identified during the postoperative follow-up have also been reported to be associated with the duration of the treatment with IABP (Felix etal) [32].

#### IABP and peripheral circulation

By enlarge, the peripheral blood flow is determined by the pressure, resistance, length and internal friction. The balloon inflation during diastole augments the blood pressure, which in its turn increases the arterio-venous gradient and improves flow. Moreover, the inflation of the balloon at diastole causes displacement of blood volume and thus the activation of the aortic barro-receptors inhibits the medullary vasoconstrictor reflex.

Peripheral resistance is reduced, resulting in the improvement of the blood flow.

Landreneau et al [33] studied the splanchic blood flow response to intra aortic balloon pump assist of prolonged hemorrhagic shock. They concluded that the intra aortic pump during the hemorrhagic shock seems to improve the vasokinetic control of the splanchnic blood flow, by eliminating the effect of hyperaemic reperfusion and thus resulting in less reperfusion injury.

Hilberman et al [34] studied the impacts of the intra aortic pump on the postoperative renal function in humans. They showed a postoperative improvement of the renal reperfusion in humans during counterpulsation.

Swartz et al [35] studied the effect of perirenal balloon placement on renal blood flow. They found a mean reduction of 66% while the balloon was in the renal site. Rastan et al [36] using CT scans identified IABP malposition to be a common finding. "Anatomic to balloon" length miss-match was found in 68.2% of the cases, with subsequently severe adverse effects. Finally, clinical reports of intra-abdominal ischemia due to "anatomic-to-balloon" device length mismatch are reported but limited [37].

## Conclusion

Due to the nature of the IABP the main complications are related to vascular injury, with studies suggesting overall angio-ischaemic complications of between 8-18% with major limb ischaemia reported to be less than 1% [38].

In general, embolic complications are said to be associated mainly with the insertion process and less with pumping, or removal. On the contrary, bacterial infections and thrombotic complications are mainly related to the actual time of the treatment with the intra aortic balloon devise.

Historically support with IABP has been associated with high complication rate, however newer studies [39] have shown low incidence of major adverse events. Physicians therefore should have little reluctance to use IABP in high-risk patients.

## Competing interests

The authors declare that they have no competing interests.

## Authors' contributions

HP carried out the literature research, participated in the sequence alignment and drafted the manuscript, AS helped with the collection of the data and the construction of the manuscript,

BA assist in data analysis, and also the development of the manuscript and advised on valuable amendments. The authors read and approved the final manuscript.
